# Fluoroscopy Time as a New Predictor of Short-Term Outcomes after Transcatheter Aortic Valve Replacement

**DOI:** 10.3390/jcdd10110459

**Published:** 2023-11-13

**Authors:** Alessandro Cafaro, Francesco Spione, Osvaldo Burattini, Daniele De Feo, Alessandro Xhelo, Chiara Palmitessa, Maurizio D’Alessandro, Vincenzo Pio Amendola, Flavio Rimmaudo, Andrea Igoren Guaricci, Alessandro Santo Bortone, Vincenzo Pestrichella, Gaetano Contegiacomo, Tullio Tesorio, Giuseppe Colonna, Fortunato Iacovelli

**Affiliations:** 1 Division of Cardiology, “V. Fazzi” Hospital, 73100 Lecce, Italy; giuseppe.colonna@tin.it; 2 Interventional Cardiology Service, “Montevergine” Clinic, GVM Care & Research, 83013 Mercogliano, Italy; francesco.spio@gmail.com (F.S.); tulliotesorio@gmail.com (T.T.); 3 Division of Cardiology, Department of Advanced Biomedical Sciences, University of Naples “Federico II”, 80131 Naples, Italy; 4 Division of Cardiology, “SS. Annunziata” Hospital, 74121 Taranto, Italy; osvaldoburattini@gmail.com (O.B.); fortunato.iacovelli@gmail.com (F.I.); 5 Division of University Cardiology, Cardiothoracic Department, Policlinico University Hospital, 70124 Bari, Italy; daniele.df93@gmail.com (D.D.F.); alessandroxhelo2@gmail.com (A.X.); chiara.palmy@gmail.com (C.P.); maurizio.dalessandro23@gmail.com (M.D.); vincenzopioamendola@icloud.com (V.P.A.); andrea.guaricci@gmail.com (A.I.G.); 6 Division of Cardiology, “Vittorio Emanuele” Hospital, 93012 Gela, Italy; rimmaudoflavio@gmail.com; 7 Division of University Heart Surgery, Cardiothoracic Department, Policlinico University Hospital, 70124 Bari, Italy; alessandrosanto.bortone@uniba.it; 8 Interventional Cardiology Service, “Mater Dei” Hospital, 70125 Bari, Italy; vpestrichella@yahoo.it; 9 Interventional Cardiology Service, “Anthea” Clinic, GVM Care & Research, 70124 Bari, Italy; gconteg@gmail.com

**Keywords:** aortic stenosis, transcatheter aortic valve replacement, fluoroscopy, Valve Academic Research Consortium, technical success, device success, early safety

## Abstract

Background: Transcatheter aortic valve replacement (TAVR) is an almost totally cine-fluoroscopic guided procedure. The amount of radiation used during the procedure is strictly related to the fluoroscopy time (FT), that has already been demonstrated to be associated with outcomes and complexity of coronary procedures. The aim of our study is to demonstrate the relationship between FT and the short-term outcomes after TAVR defined by to the Valve Academic Research Consortium (VARC)-2 and -3 consensus documents. Methods: After splitting 1797 consecutive patients into tertiles of FT, the composite endpoint early safety (ES) was adjudicated according to VARC-2 and VARC-3 definitions, whereas the composite endpoints device success (DS) and technical success (TS) according to VARC-3 criteria. Results: The absence of all these outcomes (VARC-2 ES amd VARC-3 TS, DS, and ES) was significantly associated with longer FT: this association was independent from both intraprocedural complications and other intraprocedural factors linked to longer FT, and still persisted after propensity score matching analysis. Notwithstanding, after receiver operating characteristic analysis, FT had adequate diagnostic accuracy in identifying the absence of only VARC-3 TS and VARC-2 ES. Conclusion: Longer FT is related with periprocedural and short-term outcomes after the procedure, especially in those that are more challenging. A FT duration of more than 30 min has an adequate accuracy in identifying VARC-3 technical failure (TS and DS) and absence of VARC-2 ES, selecting patients who are likely to take advantage from more careful in-hospital follow-up.

## 1. Introduction

Transcatheter aortic valve replacement (TAVR) is an established treatment option for patients with aortic valve stenosis at high surgical risk or considered unsuitable for conventional surgical aortic valve replacement (SAVR). Multiple observational and randomized clinical trials have demonstrated the feasibility and efficacy of this treatment [1,2,3,4,5,6,7,8,9,10]. Notwithstanding, recent randomized trials have shown that this percutaneous technique is not inferior to SAVR in patients at intermediate or low surgical risk [11,12,13,14,15].

TAVR is an almost totally cine-fluoroscopy-guided procedure, and the amount of radiation used is potentially dangerous for both operators and patients because of its stochastic and deterministic adverse effects [16,17]. The mean TAVR radiation dose (RD), which is strictly related to fluoroscopy time (FT) and procedure length, has been demonstrated to be similar to other percutaneous coronary interventions (PCI) of moderate complexity [18,19,20]. However, FT and RD are different parameters: FT is independent of tissue impedance, angiographer technical characteristics, and patients’ biometric parameters, while RD widely varies according to body mass index [21].

To date, no study has investigated the association between FT and short-term prognosis after TAVR. In particular, in the Valve Academic Research Consortium (VARC) consensus documents [22,23], device success (DS) and early safety (ES) are short-term composite endpoints. DS combines the absence of procedural mortality, the correct positioning of a single prosthetic heart valve into the proper anatomical location, and the intended performance of the prosthetic heart valve. ES combines the 30-day all-cause mortality, all types of stroke, life-threatening bleeding, stage 2 or 3 acute kidney injury, coronary artery obstruction requiring intervention, and valve-related dysfunction requiring another aortic valvular procedure within 30 days after TAVR. The VARC-3 consensus document has added the endpoint technical success (TS), which is a composite of freedom from mortality, successful access, the delivery of the device and retrieval of the delivery system, the correct positioning of a single prosthetic heart valve into the proper anatomical location, and the freedom from surgery or intervention related to the devices or a major vascular or access-related, or cardiac structural complications at the exit from procedure room [23]. With respect to ES definition, the VARC-3 document added other adverse events that significantly impact short- and long-term prognosis, such as cardiac structural complications, significant (moderate-to-severe) aortic regurgitation, and new permanent pacemaker implantation [22]. Finally, the definition of DS in the VARC-3 document added TS among the other endpoints included in the VARC-2 definition [22,23].

Our study aims to evaluate for the first time, in a large population, the relationship between FT and short-term outcomes after TAVR (ES according to VARC-2, and TS, DS, and ES according to VARC-3).

## 2. Materials and Methods

### 2.1. Study Population

This multicenter observational study assessed all consecutive patients who underwent TAVR at five southern Italy heart centers (“V. Fazzi” Hospital of Lecce, “Montevergine” Clinic of Mercogliano, and Policlinico University Hospital, “Anthea” Clinic, and “Mater Dei” Hospital of Bari) involved in the “Magna Graecia” TAVR registry.

Between March 2011 and April 2023, 1797 consecutive patients (785 males, mean age 80.86 ± 5.71 years, 1703 transfemoral access) suitable for TAVR were enrolled. All patients underwent preprocedural assessment with transthoracic echocardiography, coronary angiography, computed tomography (CT) scan of the heart, aorta, and peripheral arteries, carotid artery ultrasonography, and multidisciplinary evaluation by the Heart Team. The majority of the procedures were performed in a standard cardiac catheterization laboratory with the support of anesthesiology and surgical back-up by experienced operators. The devices used were balloon-expandable (*Sapien XT*, *Sapien 3*, *and Sapien 3 Ultra*, Edwards Lifesciences Inc., Irvine, CA, USA; *Myval*, Meril, Gujarat, India), self-expanding (*CoreValve*, *Engager*, *Evolut R*, *Evolut PRO and Evolut PRO+*, Medtronic, Minneapolis, MN, USA; *Portico and Navitor*, Abbott Medical, Santa Clara, CA, USA) and others (*Lotus*, *Acurate and Acurate Neo*, Boston Scientific Corporation, Marlborough, MA, USA; *Direct Flow*, Direct Flow Medical Inc., Santa Clara, CA, USA; *JenaValve*, Jenavalve, Irvine, CA, USA).

Each participating site collected all baseline demographics, clinical, laboratory, echocardiographic, surgical risk scores, intraprocedural and postprocedural data, in-hospital outcomes, and 1-month follow-up outcomes, in the same dedicated archiving software. All the adverse events as well as TS, DS, and ES composite endpoints were also re-adjudicated retrospectively, by an external committee of interventional cardiologists, according to both VARC-2 and VARC-3 criteria [22,23]. All TAVR-related complications (according to VARC-2 and VARC-3 definitions, both separately and then globally considered) were divided into intra- and postprocedural complications. The time delay between the end of the TAVR procedure and the first postprocedural complication occurrence was also registered.

The patient population was retrospectively divided according to FT (minutes) tertiles and then based on enrollment-time tertiles in order to analyze FT and RD trends during TAVR learning curves.

### 2.2. Statistical Analysis

Statistical analysis was performed using SigmaStat 3.5, SPSS 25.0, and STATA 13.0 softwares. Continuous variables were expressed as the mean ± standard deviation and median (interquartile ranges) of absolute numbers; categorical variables were expressed as frequencies and percentages. As appropriate, comparisons were performed using a *t*-test, the Mann–Whitney U test, one-way ANOVA, ANOVA on ranks, Fisher’s exact test, or χ^2^ test. Pairwise multiple comparisons after ANOVAs were conducted using Holm–Sidak or Dunn’s test as properly indicated by definitions. The normal distribution was assessed with Kolmogorov–Smirnov tests. A receiver-operating characteristic (ROC) curve analysis was performed in order to establish the threshold levels of FT that provided the best cut-off for the absence of ES according to VARC-2 and VARC-3 definitions. Area under the curve (AUC) values were calculated with confidence intervals (CIs) through concordance statistics to measure test accuracy. The DeLong test was used to identify AUC standard errors. The calibration of FT was evaluated by comparing the mean predicted probability and the mean observed frequency of absence of ES with goodness-of-fit R-squared and Cochran–Armitage tests, calibration plots, and the estimation of a calibration slope. After this, new optimal cut-off points for the absence of ES were selected using Youden’s tests, reporting Youden’s indexes: we evaluated sensitivity and specificity according to these new cut-off points. The relationship between FT and the absence of ES was also analyzed after propensity score matching (PSM) including as covariates those factors that were considered to increase the time of the procedure: pre-TAVR ejection fraction, maximum transvalvular gradient, vascular access other than the femoral one (e.g., transapical and direct aortic access), prosthesis predilatation, self-expanding valve implantation, postdilatation, and intraprocedural complications. All statistical tests were two-sided. For all tests, a *p*-value <0.05 was considered statistically significant.

## 3. Results

### 3.1. Baseline Characteristics

The patient population was divided according to FT (minutes) tertiles: for the first group, 13.94 ± 2.93 min; for the second group, 21.31 ± 1.99 min; and for the third group, 38.31 ± 18.83 min.

All clinical and preprocedural data of the study population are shown in Table 1.

No statistically significant differences were found in terms of preprocedural characteristics like patients’ characteristics, previous cardiovascular history, comorbidities, and mortality risk scores. Only echocardiographic parameters like left ventricular ejection fraction (*p* < 0.001) and maximum aortic gradient (*p =* 0.032) were mildly but significantly different between the three groups.

### 3.2. Procedural Characteristics

All procedural and postprocedural data, and outcomes are shown in Table 2.

Some procedural details, namely transfemoral access (*p* = 0.005), predilatation (*p* < 0.001), self-expanding bioprosthesis (*p* = 0.011), valve size >26 mm (*p* = 0.048), postdilatation (*p* < 0.001), contrast mean (CM) amount (*p* < 0.001), and RD (*p* < 0.001), were significantly associated with FT.

With respect to complications according to VARC-3 criteria, FT was significantly associated with bleedings (*p* < 0.001), transfusions (*p* < 0.001), vascular complications (*p <* 0.001), percutaneous closure device failure (*p =* 0.005), cardiac arrest during the procedure (*p <* 0.001), and acute myocardial infarction (*p =* 0.009). FT results were also significantly linked (*p* = 0.016) with postprocedural complications, and patients in the longest FT group experienced a complication earlier than those with shorter FT (*p* = 0.049). Moreover, longer hospitalizations were significantly associated with higher FT during the TAVR procedure (*p* < 0.001).

**Figure 1:**
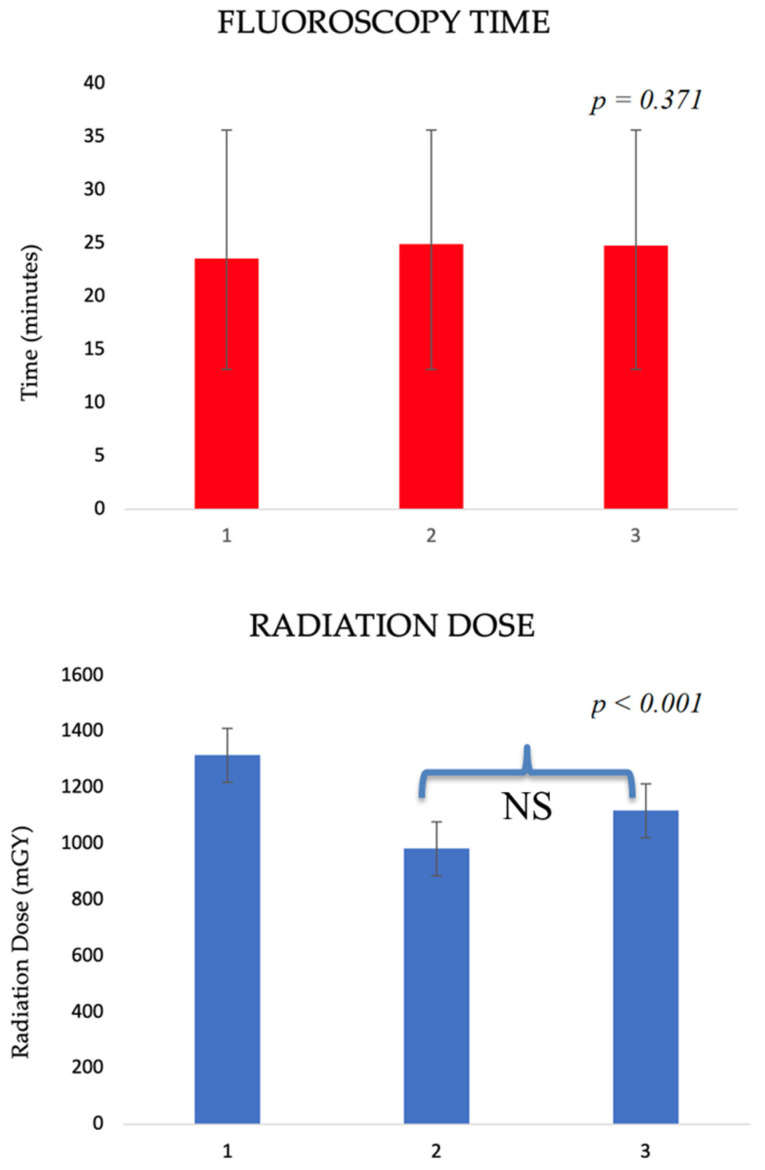
TAVR learning curve. Fluoroscopy time and radiation dose (mean and standard deviation) according to TAVR enrollment time tertiles (1st tertile: 598 patients from April 2011 to September 2017; 2nd tertile: 600 patients from October 2017 to November 2020; 3rd tertile: 599 patients from December 2020 to April 2023). NS: not significant.

Figure 1 shows the variation in FT and RD after splitting the population into tertiles according to the period of enrollment. There was no significant difference in FT across the tertiles of enrollment time (23.54 ± 15.41; 24.92 ± 18.13; and 24.74 ± 12.01 min; *p* = 0.371). On the other hand, there was a significant variation in RD that spanned along the study time (*p* < 0.001). RD significantly decreased between the first and the second enrolling time tertile, while the slight RD increase between the second and the third tertiles was not significant after pairwise comparisons (1143.96 ± 82.72 vs. 1449.70 ± 73.23 mGy; *p* = 0.175).

### 3.3. Outcomes

Table 2 also shows outcomes’ incidence and its relationship with FT. Concerning the outcomes defined by VARC-3 criteria, higher FT was significantly associated with lower TS, DS and ES (*p <* 0.001, *p =* 0.021, and *p =* 0.013, respectively). Also considering VARC-2 criteria, the absence of ES was significantly associated with FT (*p <* 0.001). The global rates of VARC-2 and VARC-3 postprocedural complications significantly increased (*p <* 0.016) across FT tertiles. Furthermore, Table 3 reveals the association between FT and outcomes, defined as TS, DS, and ES according to VARC-3 criteria and ES according to VARC-2 criteria, after PSM: in fact, higher FT was still significantly associated with the absence of TS (*p =* 0.001), DS (*p <* 0.001), and ES (*p* = 0.035), according to VARC-3 criteria and with the absence of ES (*p =* 0.046) according to VARC-2 criteria.

**Figure 2:**
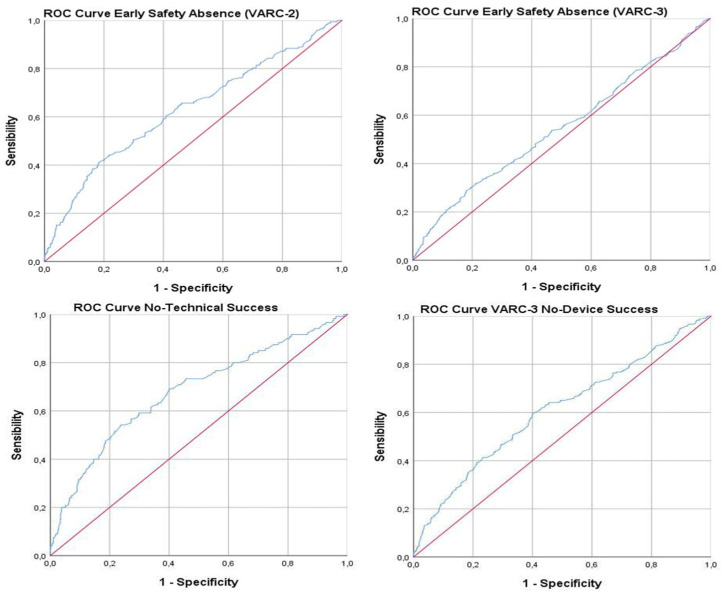
Absence of TS, DS, and ES according to FT–ROC curve analysis.

Finally, the ROC analysis showed a significant correlation between FT and these outcomes: VARC-3 TS (AUC 0.680, 95% CI 0.654–0.704, sensitivity 54.17%, specificity 76.21%, *p* < 0.001), VARC-3 DS (AUC 0.608, 95% CI 0.581–0.633, sensitivity 60.56%, specificity 57.89%, *p* < 0.001), VARC-3 ES (AUC 0.545, 95% CI 0.518–0.571, sensitivity 29.5%, specificity 80%, *p* = 0.008), and VARC-2 ES (AUC 0.628, 95% CI 0.601–0.654, sensitivity 41.28%, specificity 81.53%, *p* < 0.001) (Table 4, Figure 2). Nevertheless, based on the AUC of the cut-off values established with the highest Youden’s indexes, good performance was observed only in the detection of VARC-3 TS (cut-off 27.8 ± 0.04 min) and VARC-2 ES (cut-off 30.1 ± 0.03 min).

## 4. Discussion

The main findings of our study can be summarized as follows: (1) as expected, longer FT during TAVR is related to more challenging procedures; (2) short-term outcomes after TAVR are related to FT, also after PSM that balances intraprocedural complications and all time-consuming procedural features, so FT independently predicts postprocedural complications; (3) the cut-offs identified after ROC analysis have sufficient accuracy to detect VARC-3 TS and VARC-2 ES; (4) the variation in FT over time is not significantly related to the TAVR learning curve.

Our study is the first that investigated, in a large TAVR cohort, the relationship between FT and short-term outcomes, after the last updated VARC-3 consensus document [23]. Previous studies demonstrated that prolonged FT is associated with PCI lesions’ complexity [24,25,26]. In our analysis, longer FT was associated with transfemoral access approach and more challenging procedures: need for predilatation and postdilatation procedures, self-expanding valve implantation, higher CM amount used, and higher RD generated during the procedure. However, the higher FT length when transfemoral access was used instead of a surgical approach (like transapical and transsubclavian) is easily explained by the fact that in our study, the percutaneous access management was in the vast majority of procedures completely cine-fluoroscopy-guided. Currently, ultrasound-guided (USG) femoral puncture is widely performed. The USG femoral access in our study involved a slight minority of TAVR procedures (only 46 patients with USG puncture performed both on main and side femoral accesses). Thus, we could assume that our results probably would have been even more consistent with a wider usage of USG, because of less fluoroscopy use during standard uncomplicated procedures. Moreover, bleeding and complication rates are different between transfemoral and other TAVR accesses [27]. Consequently, all those variables were balanced using PSM.

To date, no study has analyzed the relationship between FT and short-term outcomes in TAVR. Only radiation exposure during the procedure has been investigated and has been shown to be comparable to PCI of moderate complexity [18,19,20]. Interestingly, in our study, the absence of all short-term composite outcomes (VARC-3 TS, DS, and ES, and VARC-2 ES) was significantly associated with longer FT, and this association persisted after PSM, also considering as covariates all the variables that could influence the length of the procedure, including intraprocedural complications. FT appeared to be an independent predictor of short-term TAVR-related postprocedural complications. This could be explained by the fact that in interventional cardiology, the shorter the duration of the procedures, the fewer the complications and consequently the better the outcomes.

However, although the ROC analysis showed a significant correlation between FT and these outcomes, the identified cut-offs do not have adequate diagnostic accuracy in adjudicating DS and ES according to VARC-3 criteria, due to the fact that their AUC was never above 0.6. Conversely, the cut-offs of 27.8 ± 0.04 min for VARC-3 TS, and of 30.1 ± 0.03 for VARC-2 ES have adequate diagnostic accuracy. These values show that when the fluoroscopy lasted more than 30 min, it is more likely that the patient experienced technical failure at the exit from the catheterization laboratory or complications in the short term.

The reason why this cut-off was able to predict the absence of VARC-2 ES rather than VARC-3 ES could properly be explained by the possible limits of new VARC-3 criteria, namely the more the number of complications included in this composite endpoint, the more the decline in its diagnostic performance. Indeed, as previously reported, even the accuracy of the mortality risk score is lower with VARC-3 than with VARC-2 criteria [28]. One other reason why longer FT independently predicted adverse events after TAVR is that this simple parameter probably takes into account many procedural features that universally make TAVR procedures more challenging and long-lasting. For example, horizontal annulus angle, marked ascending aorta slope, unfavourable aortic arch type, aorto-ilio-femoral stenosis, calcification and tortuosity, valve crossing time, usage of different rapid pacing modalities, could be all factors that no score measured around the globe might consider simultaneously. All those parameters could cause longer FT and hard maneuvers linked with possible delayed cerebral events (for debris micro-embolization) or with post-TAVR bleeding and vascular complications. Careful CT scan planning, also implemented with score analysis such as the recently published “Hostile Score” [29,30], could reduce FT, RD, and CM usage during TAVR. However, we did not detect any significant correlation between CM amount during CT and RD during TAVR procedure (*p* = 0.567).

Finally, in terms of the TAVR learning curve (Figure 1), there was no significant variation in FT, while the reduction in RD administered during the period of enrollment was significant only between the first and the second tertile of the enrollment period. This finding could be explained by the fact that the first tertile of enrollment, covering about 7 years, spanned for a longer time than the second and the third ones. Thus, the first tertile of enrollment constitutes most of the learning curve. The RD reduction is likely related to technological advancements of the newer radiological angiographers. Another explanation is that the majority of invasive coronary angiography procedures were performed simultaneously with TAVR in the third tertile of the enrollment period. Thus, even though the operators improved their skills, there was no decrease in FT or RD between the second and third tertile of TAVR enrollment, due to the fact that more procedures were performed at the same time. However, our data showed no significant increase in the revascularization rate during TAVR according to FT tertiles, so an indirect link between longer FT and myocardial injury related to myocardial revascularization during TAVR could be excluded. Although there is no other way to manage challenging anatomy and procedure-related adverse events without precise fluoroscopic guiding, and so reducing FT is not a reasonable objective, our results could highly impact daily practice during postprocedural follow-up. FT could be considered a simple parameter to independently predict short-term composite outcomes after TAVR procedures: if longer than 30 min, it could be useful to detect patients who need a more careful follow-up and who therefore cannot be included in fast-track and discharge programs.

## 5. Limitations

Although data were obtained from a prospectively collected database, this is an unspecified post hoc analysis. Therefore, we cannot exclude that potential confounding factors not considered in the model may have influenced the results. The effect of a learning curve and changes in treatment strategy is also heterogeneous, as the study spanned more than a decade. Furthermore, we believe that aspects of management that were not controlled or specified may have been a source of bias. Finally, an independent committee did not adjudicate all clinical events that were site-referred.

## 6. Conclusions

This is the first study demonstrating that longer FT is related to periprocedural complications and the absence of short-term composite outcomes after TAVR, especially in more challenging procedures. A FT duration of more than 30 min has adequate accuracy in identifying VARC-3 technical failure and the absence of VARC-2 ES. Our results demonstrate how FT, a simple parameter that can be easily collected after a procedure, is influenced by many procedural features that are difficult to individually analyze. Therefore FT can help to select those patients who need careful follow-up and who could benefit from longer hospital stay in order to prevent and treat complications.

## Figures and Tables

**Table 1:** Baseline characteristics of the study population according to FT tertiles.

Variable	All	Fluoroscopy Time			
		1st	2nd	3rd	*p*
***Patients characteristics***					
Age (years)	80.86 ± 5.71	80.72 ± 5.59	80.61 ± 6.13	81.17 ± 5.48	0.403
Male	785/1797 (43.68%)	206/491 (41.95%)	210/438 (47.94%)	220/463 (47.52%)	0.118
Body mass index (kg/m^2^)	27.34 ± 4.73	27.34 ± 4.77	27.20 ± 4.32	27.48 ± 5.07	0.802
Hypertension	1690/1785 (94.68%)	453/486 (93.21%)	409/437 (93.59%)	433/457 (94.75%)	0.597
Diabetes mellitus	577/1788 (32.27%)	160/488 (32.79%)	149/437 (34.10%)	154/458 (33.62%)	0.912
Insulin	238/1759 (13.53%)	65/483 (13.46%)	71/424 (16.74%)	72/447 (16.11%)	0.339
Dyslipidemia	1170/1786 (65.51%)	320/487 (65.71%)	296/437 (67.73%)	304/457 (66.52%)	0.807
Smoking	122/1744 (6.99%)	40/483 (8.28%)	23/421 (5.46%)	28/436 (6.42%)	0.227
Anemia	977/1782 (54.83%)	254/490 (51.84%)	239/435 (54.94%)	267/459 (58.17%)	0.147
COPD	452/1786 (25.31%)	122/487 (25.01%)	111/437 (25.40%)	138/458 (30.13%)	0.151
Neurological dysfunction	146/1759 (8.30%)	36/483 (7.45%)	35/427 (8.20%)	35/451 (7.76%)	0.916
Severe liver disease	41/1783 (2.30%)	13/488 (%)	10/435 (%)	10/457 (%)	0.882
PAD	421/1758 (23.95%)	128/483 (26.50%)	90/428 (21.03%)	109/451 (24.17%)	0.155
Carotid stenosis ≥ 50%	45/1354 (3.23%)	6/297 (%)	10/338 (%)	15/345 (%)	0.235
Critical preoperative state	66/1779 (3.71%)	18/485 (3.71%)	23/435 (5.29%)	18/456 (3.95%)	0.454
CAD history	448/1784 (25.11%)	125/488 (25.61%)	109/436 (25.00%)	130/456 (28.51%)	0.441
Prior myocardial infarction	255/1786 (14.28%)	76/488 (15.57%)	64/436 (14.68%)	86/457 (18.82%)	0.208
Prior cardiac surgery	253/1787 (14.16%)	68/489 (13.91%)	68/437 (15.56%)	72/458 (15.72%)	0.687
Prior myocardial revascularization	406/1791 (22.68%)	119/489 (24.33%)	95/437 (21.74%)	118/459 (25.71%)	0.370
PCI	248/1791 (13.85%)	76/489 (15.54%)	48/437 (10.98%)	74/459 (16.12%)	0.056
CABG	92/1791 (5.14%)	28/489 (5.73%)	27/437 (6.18%)	26/459 (5.66%)	0.938
PCI + CABG	66/1791 (3.69%)	15/489 (3.07%)	20/437 (4.58%)	18/459 (3.92%)	0.486
Myocardial revascularization close to TAVR	271/1791 (15.13%)	67/486 (13.79%)	54/437 (12.36%)	65/463 (14.04%)	0.728
PCI	266/1791 (14.85%)	66/486 (13.58%)	53/437 (12.13%)	62/463 (13.39%)	0.781
CABG	4/1791 (0.22%)	1/486 (0.21%)	1/437 (0.23%)	2/463 (0.43%)	0.778
PCI + CABG	1/1791 (0.05%)	0/486 (0.00%)	0/437 (0.00%)	1/463 (0.22%)	0.369
Residual significant CAD during TAVR	214/1782 (12.01%)	60/483 (12.42%)	48/437 (12.34%)	49/459 (10.67%)	0.666
Prior PM/ICD/CRT implantation	222/1772 (12.53%)	57/485 (11.75%)	49/433 (11.32%)	62/454 (13.66%)	0.522
NYHA functional class III-IV	1475/1786 (82.59%)	402/487 (82.55%)	355/437 (81.24%)	356/457 (77.90%)	0.181
CKD	743/1797 (41.35%)	212/491 (43.18%)	178/438 (40.64%)	200/463 (43.20%)	0.671
***Electrocardiography***					
Sinus rhythm	1462/1788 (81.77%)	390/488 (79.92%)	354/437 (81.01%)	383/458 (83.62%)	0.325
Atrial fibrillation/flutter	326/1788 (18.23%)	98/488 (20.08%)	83/437 (18.99%)	75/458 (16.38%)	0.325
PM-induced rhythm	94/1788 (5.26%)	31/488 (6.35%)	24/437 (5.49%)	24/458 (5.24%)	0.741
***Echocardiography***					
LVEF (%)	53.345 ± 10.21	54.22 ± 10.81	52.88 ± 10.47	52.33 ± 9.77	<0.001
Maximum aortic gradient (mmHg)	75.67 ± 21.22	73.43 ± 20.45	77.38 ± 20.00	76.33 ± 22.69	0.032
Mean aortic gradient (mmHg)	46.40 ± 14.33	45.27 ± 14.29	47.21 ± 13.23	46.92 ± 14.93	0.074
Moderate-to-severe mitral regurgitation	679/1678 (40.46%)	175/455 (38.46%)	179/407 (43.98%)	194/429 (45.22%)	0.095
Pulmonary arterial systolic pressure (mmHg)	40.22 ± 13.37	40.23 ± 12.47	39.72 ± 12.84	40.07 ± 12.83	0.834
***Mortality risk scores***					
Logistic EuroSCORE	16.14 ± 12.31	16.04 ± 12.76	15.94 ± 12.18	17.49 ± 13.55	0.068
EuroSCORE II	5.79 ± 12.75	5.61 ± 5.84	5.26 ± 5.35	6.04 ± 6.79	0.283
STS-PROM	4.60 ± 3.55	4.70 ± 3.60	4.47 ± 3.45	4.96 ± 4.32	0.077
STS-PROM ≥ 8	176/1779 (9.90%)	45/486 (9.26%)	44/435 (10.11%)	60/453 (13.24%)	0.122

COPD = chronic obstructive pulmonary disease; PAD = peripheral artery disease; CAD = coronary artery disease; PCI = percutaneous coronary intervention; CABG = coronary artery by-pass grafting; TAVR = transcatheter aortic valve replacement; PM = pacemaker; ICD = implantable cardioverter-defibrillator; CRT = cardiac resynchronization therapy; NYHA = New York Heart Association; CKD = chronic kidney disease; LVEF = left ventricular ejection fraction; EuroSCORE = European System for Cardiac Operative Risk Evaluation; STS-PROM = Society of Thoracic Surgery predictive risk of mortality.

**Table 2:** Procedural features and outcomes according to FT tertiles.

Variable	All	Fluoroscopy Time			
		1st	2nd	3rd	*p*
***Procedural details***					
Transfemoral access route	1703/1797 (94.77%)	444/491 (90.43%)	416/438 (94.98%)	440/463 (95.03%)	0.005
Other access routes	94/1797 (5.23%)	47/491 (9.57%)	22/438 (5.02%)	23/463 (4.97%)	0.005
Trans-subclavian	27/1797 (1.57%)	9/491(1.83%)	6/438 (1.37%)	12/463 (2.59%)	0.404
Transapical	57/1797 (3.17%)	36/491 (7.33%)	10/438 (2.28%)	9/463 (1.94%)	<0.001
Direct aortic	8/1797 (0.44%)	1/491(0.20%)	6/438 (1.37%)	1/463 (0.22%)	0.029
Orotracheal intubation	233/1796 (12.97%)	73/491 (14.87%)	66/437 (15.10%)	90/463 19.44%)	0.107
Valve-in-valve	73/1794 (4.07%)	16/491 (3.26%)	20/437 (4.58%)	27/461 (5.86%)	0.157
Predilatation	827/1784 (46.36%)	186/489 (38.04%)	248/436 (56.88%)	282/456 (61.84%)	<0.001
Valve kind					
Balloon-expandable	551/1797 (30.66%)	190/491 (38.70%)	153/438 (34.93%)	130/463 (28.08%)	0.002
Self-expanding	1124/1797 (62.55%)	266/491 (54.17%)	248/438 (56.62%)	294/463 (63.50%)	0.011
Others	122/1797 (6.79%)	35/491 (7.13%)	37/438 (8.45%)	39/463 (8.42%)	0.691
Valve Size > 26 mm	722/1793 (40.27%)	175/489 (35.79%)	166/438 (37.90%)	200/461 (43.38%)	0.048
Postdilatation	479/1795 (26.68%)	97/491 (19.76%)	112/436 (25.69%)	145/463 (31.32%)	<0.001
CM volume (mL)	149.97 ± 76.36	130.431 ± 54.03	161.14 ± 67.22	197.61 ± 96.94	<0.001
Radiation dose (mGy)	1366.18 ± 1241.57	1070.68 ± 1051.40	1381.24 ± 1070.11	2112.15 ± 1748.60	<0.001
***Complications and outcomes (VARC-3)***					
AKI	272/1714 (15.87%)	70/472 (14.83%)	50/420 (11.90%)	72/433 (16.63%)	0.142
CVVH	41/1730 (2.37%)	10/476 (2.10%)	8/420 (1.90%)	15/438 (3.42%)	0.288
Chronic hemodialysis	9/1667 (0.54%)	3/460 (0.65%)	2/409 (0.49%)	4/423 (0.95%)	0.724
Bleeding (VARC-3)	588/1399 (48.03%)	124/343 (36.15%)	139/313 (44.41%)	214/362 (59.12%)	<0.001
Type 1	192/ (13.72%)	34/343 (9.91%)	55/313 (17.57%)	72/362 (19.89%)	<0.001
Type 2	307/1399 (21.94%)	67/343 (19.53%)	67/313 (21.41%)	102/362 (28.18%)	0.017
Type 3–5	89/1399 (6.36%)	23/343 (6.71%)	17/313 (5.43%)	40/362 (11.05%)	0.016
BARC ≥ 3	561/1773 (32.37%)	124/477 (26.00%)	117/424 (27.59%)	208/449 (46.32%)	<0.001
Need of transfusion	298/1721 (17.31%)	61/475 (12.84%)	65/419 (15.51%)	108/442 (24.43%)	<0.001
1 unit	140/1721 (8.13%)	30/475 (6.32%)	31/419 (7.40%)	52/442 (11.76%)	0.008
2 units	106/1721 (6.16%)	23/475 (4.84%)	23/419 (5.49%)	38/442 (8.60%)	0.046
>2 units	52/1721 (3.02%)	8/475 (1.68%)	11/419 (2.62%)	18/442 (4.07%)	0.086
Vascular complications	286/1765 (16.20%)	59/484 (12.19%)	57/436 (13.07%)	118/455 (25.93%)	<0.001
minor	170/1765 (9.63%)	41/484 (8.47%)	34/436 (7.80%)	65/455 (14.29%)	0.002
major	116/1765 (6.57%)	18/484 (3.72%)	23/436 (5.27%)	53/455 (11.65%)	<0.001
Access-site related vascular complications	224/339 (66.08%)	22/69 (31.88%)	43/86 (50.00%)	47/137 (34.31%)	0.029
PCD failure	101/1556 (6.49%)	20/404 (4.72%)	22/373 (5.90%)	40/387 (10.34%)	0.005
At least moderate residual aortic Regurgitation	129/1562 (8.26%)	26/412 (6.31%)	30/377 (7.96%)	38/398 (9.55%)	0.223
Permanent PM implantation	226/1572 (14.38%)	51/419 (12.17%)	64/484 (16.67%)	60/381 (15.75%)	0.163
ECM/cardiac arrest	66/1713 (3.85%)	15/473 (3.17%)	7/425 (1.65%)	34/440 (7.73%)	<0.001
New-onset atrial fibrillation/flutter	124/1412 (8.78%)	33/375 (8.80%)	25/343 (7.29%)	33/376 (8.78%)	0.707
Acute myocardial infarction	19/1774 (1.07%)	2/486 (0.41%)	3/437 (0.69%)	11/454 (2.42%)	0.009
Stroke/TIA	34/1773 (1.92%)	6/487 (1.23%)	8/437 (1.83%)	7/453 (1.54%)	0.759
Hospital length of stay (days)	5.73 ± 9.63	4.99 ± 3.52	5.53 ± 3.93	6.34 ± 4.46	<0.001
Hospital length of stay > 5 days	627/1722 (36.41%)	139/472 (29.45%)	160/424 (37.74%)	194/431 (45.01%)	<0.001
Technical success	1612/1752 (92.01%)	456/480 (95.00%)	406/431 (94.20%)	382/453 (84.33%)	<0.001
Device success	1562/1764 (88.55%)	442/485 (91.13%)	383/431 (88.86%)	385/451 (85.37%)	0.021
Periprocedural mortality	43/1734 (2.48%)	10/475 (2.10%)	10/423 (2.36%)	19/452 (4.21%)	0.118
Mortality at one year (F-U)	59/1686 (3.50%)	19/465 (4.09%)	10/413 (2.42%)	20/432 (4.63%)	0.212
Early safety absence (VARC-2)	222/1732 (12.82%)	42/477 (8.80%)	43/426 (10.09%)	87/449 (19.38%)	<0.001
Early safety absence (VARC-3)	559/1732 (32.27%)	141/477 (29.56%)	115/426 (26.99%)	161/449 (35.86%)	0.013
Postprocedural complications (VARC-2 and VARC-3)	427/839 (50.89%)	91/203 (44.83%)	100/188 (53.19%)	146/250 (58.40%)	0.016
Complication time delay (days) from TAVR	3.44 ± 39.39	3.13 ± 18.55	6.99 ± 19.80	2.55 ± 11.97	0.049

AKI = acute kidney injury; CVVH = continuous venovenous hemofiltration; PCD = percutaneous closure device; ECM = external cardiac massage; TIA = transient ischemic attack; F-U = follow up; VARC = Valve Academic Research Consortium.

**Table 3:** Association between fluoroscopy time and outcomes before and after propensity score matching.

**Variable**	**Technical Success**		*p*	T-Statistic
	Yes	No		
Fluoroscopy time min (unmatched)	23.82 ± 2.32	37.99 ± 2.32	<0.001	2.58
Fluoroscopy time min (matched after PSM)	22.01 ± 4.23	37.99 ± 4.23	0.001	1.90
	**Device Success (VARC-3)**		*p*	T-statistic
	Yes	No		
Fluoroscopy time min (unmatched)	23.70 ± 1.93	31.95 ± 1.93	<0.001	4.27
Fluoroscopy time min (matched after PSM)	22.83 ± 2.76	32.23 ± 2.76	0.007	3.41
	**Early Safety (VARC-2)**		*p*	T-statistic
	Yes	No		
Fluoroscopy time min (unmatched)	23.72 ± 3.05	33.72 ± 2.05	<0.001	4.87
Fluoroscopy time min (matched after PSM)	22.11 ± 3.10	33.72 ± 3.10	0.046	3.74
	**Early Safety (VARC-** **3** **)**		*p*	T-statistic
	Yes	No		
Fluoroscopy Time min (unmatched)	23.58 ± 1.80	28.23 ± 1.80	<0.001	2.58
Fluoroscopy Time min (matched after PSM)	24.09 ± 3.17	28.23 ± 3.17	0.035	1.90

PSM = propensity score matching; VARC: Valve Academic Research Consortium.

**Table 4:** ROC analysis of VARC-2 and VARC-3 outcomes according to FT.

	AUC ± DeLong Standard Error	95% CI	Asymptotic Significance	Cut-Off	Youden Index	Sensitivity (%)	Specificity (%)	Accuracy (%)	LR−/LR+	Adjusted R-Square	Slope
No technical success	0.680 ± 0.028	0.654–0.704	<0.001	27.8 ± 0.04	0.305	54.17	76.21	74.27%	0.60–2.27	0.046	0.886
No device success (VARC-2)	0.590 ± 0.024	0.564–0.616	<0.001	22 ± 0.04	0.158	58.60	56.28	56.55%	0.73–1.34	0.009	0.866
No device success (VARC-3)	0.608 ± 0.021	0.581–0.633	<0.001	22 ± 0.03	0.195	60.56	57.89	58.40%	0.78–1.44	0.026	0.948
No early safety (VARC-2)	0.628 ± 0.024	0.601–0.654	<0.001	30.1 ± 0.03	0.229	41.28	81.53	76.41%	0.72–2.23	0.032	0.929
No early safety (VARC-3)	0.545 ± 0.017	0.518–0.571	0.008	30.00 ± 0.02	0.108	29.50	80.86	65.01%	0.87–1.54	0.011	0.998

VARC = Valve Academic Research Consortium; AUC = area under the curve; CI = confidence interval.

## Data Availability

Data are accessible with the article (raw data are available upon individual request).
